# Antibiotics and Carbohydrate-Containing Drugs Targeting Bacterial Cell Envelopes: An Overview

**DOI:** 10.3390/ph15080942

**Published:** 2022-07-29

**Authors:** Federico Riu, Alessandro Ruda, Roberta Ibba, Simona Sestito, Ilenia Lupinu, Sandra Piras, Göran Widmalm, Antonio Carta

**Affiliations:** 1 Department of Medicine, Surgery and Pharmacy, University of Sassari, Via Muroni 23/A, 07100 Sassari, Italy; federicoriu1@gmail.com (F.R.); ilenialup@gmail.com (I.L.); piras@uniss.it (S.P.); acarta@uniss.it (A.C.); 2 Department of Organic Chemistry, Arrhenius Laboratory, Stockholm University, S-106 91 Stockholm, Sweden; alessandro.ruda@su.se (A.R.); goran.widmalm@su.se (G.W.); 3 Department of Chemical, Physical, Mathematical and Natural Sciences, University of Sassari, Via Vienna 2, 07100 Sassari, Italy; ssestito@uniss.it

**Keywords:** carbohydrate-based drugs, cell wall polysaccharides, bacterial membrane, antibiotic resistance, carbohydrate-based vaccines

## Abstract

Certain bacteria constitute a threat to humans due to their ability to escape host defenses as they easily develop drug resistance. Bacteria are classified into gram-positive and gram-negative according to the composition of the cell membrane structure. Gram-negative bacteria have an additional outer membrane (OM) that is not present in their gram-positive counterpart; the latter instead hold a thicker peptidoglycan (PG) layer. This review covers the main structural and functional properties of cell wall polysaccharides (CWPs) and PG. Drugs targeting CWPs are discussed, both noncarbohydrate-related (β-lactams, fosfomycin, and lipopeptides) and carbohydrate-related (glycopeptides and lipoglycopeptides). Bacterial resistance to these drugs continues to evolve, which calls for novel antibacterial approaches to be developed. The use of carbohydrate-based vaccines as a valid strategy to prevent bacterial infections is also addressed.

## 1. Introduction

### 1.1. Bacteria

#### 1.1.1. Gram-Positive and Gram-Negative Bacteria: General Remarks

**Figure 1:**
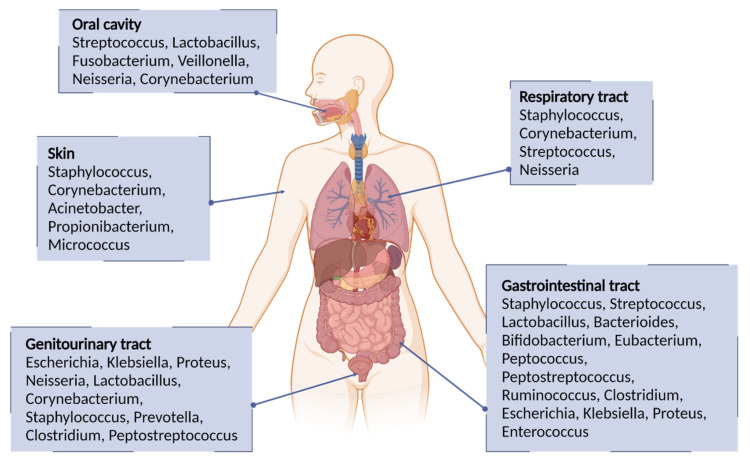
Schematic representation of target human districts of the main gram-positive and gram-negative bacteria. Created with BioRender.com (Accessed on 29 April 2022).

Bacteria are microscopic single-celled organisms that are listed among the oldest known life forms on Earth. They can be found in the soil, ocean water, ice, and underneath the earth’s crust; that is because these microorganisms are often essential to other organisms’ life [1,2,3]. The worldwide bacterial population is estimated to reach 2 × 10^30^ cells, with a major role in the ecosystem [4]. Moreover, nitrogen-fixing bacteria are essential for the natural growth of several plant species [5,6]. The bacterial fermentation process is also fundamental for the digestive systems of sheep and cattle. In the food industry area, bacteria are involved in the manufacturing of many products, including dairy products, baked goods, and alcoholic beverages [7]. In industrial microbiology, microorganisms are grown on a large scale to produce compounds such as antibiotics, enzymes, and various chemicals, while in biotechnology, genetically-modified organisms are used to synthesize, for example, human proteins for medical use. Bacteria also live in the human and animal bodies, particularly on the skin, airways, oral cavity, and the digestive, reproductive, and urinary systems, normally without causing any harm [8]. They populate the resident flora, or “microbiota”, are essential to facilitate food digestion or prevent the proliferation of other more dangerous bacteria, and acting as saprophytes [9,10,11]. Figure 1 depicts the representative genera of the constitutive bacterial flora in humans [12,13,14,15]. The intestinal microbiota plays a central role in the development of the immune system and the modulation of its function. This has led to investigations of the mechanisms that link autoimmune diseases or allergies to changes in the microbiome [16,17,18,19]. Currently, the connections between the human body and the microbiome are not limited to the gastrointestinal or immune system but concern almost all other systems. Alzheimer’s disease, multiple sclerosis, and autism have been studied for a putative role of microbiota in their development [20,21,22,23]. Based on the bacterium-host relationship, these microorganisms can be divided into four categories: symbionts, that live and multiply in contact with the host without causing damage and establishing a beneficial reciprocal relationship; diners that live and multiply in contact with the host without causing damage; pathogens that take advantage of the host causing diseases, from mild to serious ones; and opportunists, which normally are harmless but can cause disease, even serious ones if a weakening of the defense system of the organism occurs. Among them, pathogens represent a small percentage of the entire known bacterial population.

At the beginning of the 20th century, infectious diseases originating from bacterial and viral pathogens were the main cause of death. A decrease in incidence occurred through the years thanks to a greater understanding of the infectious process, the improvement of public health services, vaccination campaigns, and last but not least, the discovery of antibiotics, which made the managing of infectious diseases possible.

#### 1.1.2. Morphology of Gram-Positive and Gram-Negative Bacteria

Bacteria are unicellular organisms that are formed by a prokaryotic cell, with dimensions ranging from 0.2 to 2 µm. The cell is surrounded by the plasma membrane, which regulates the transport of nutrients and waste substances. The plasma membrane is covered by a wall with different characteristics, leading to the well-known division into gram-positive and gram-negative bacteria. In the gram-negative cell wall, a lipid bilayer membrane, the outer membrane (OM) is present, while the above-mentioned plasma membrane is the inner membrane (IM). In the inter-membrane space, there is a thin layer of peptidoglycan (or murein), referred to as the periplasm, a complex polymer that is made up of amino sugars and amino acids that completely envelop the cell. The outer membrane acts as a barrier against the diffusion of many compounds, making the cell more resistant to toxic substances. Gram-positive bacteria do not have an outer membrane and their wall is mainly composed of a thick layer of peptidoglycan [24].

The different composition of the bacterial wall determines the different coloration that is taken up by the bacteria with the dye that was created by the Danish microbiologist Hans Christian Gram in 1884 and thus allows one to discern the two groups of bacteria. Gram stain is still used in normal clinical practice, as the first step in the screening of a biological sample to identify the pathogenic microorganisms present [25].

In addition to the above-described cell wall, some bacteria possess additional layers of coating (S layer, capsule, or glycocalyx) and surface appendages (pili, fimbriae, flagella). The capsule or mucous layer consists of polysaccharide or protein substances that are secreted on the cell surface by bacteria for several functions, including adhesion (e.g., on solid surfaces generating biofilms) and protection.

#### 1.1.3. The Cytoplasmic Membrane

The cytoplasmic membrane is a thin barrier that surrounds the cell and separates the cytoplasm from the extracellular environment. The membrane is made of a phospholipid bilayer: phospholipids contain both hydrophobic (fatty acid) and hydrophilic (glycerol-phosphate) components. In the phospholipid membrane, the fatty acids point inward toward each other to form the hydrophobic environment, while the hydrophilic portion remains exposed to the external environment or the cytoplasm [26]. The membrane has a thickness of 6–8 nm and can be seen using an electron microscope: it appears as two dark-colored lines (glycerophosphate) that are separated by a lighter area (fatty acids). The structure is fluid and the proteins that are immersed in the phospholipid bilayer can undergo both rotational and lateral movement. The cytoplasmic membranes of some bacteria are strengthened by molecules called hopanoids, which are rigid planar molecules and structural analogs of sterols [27]. Many membrane proteins are firmly embedded in the membrane and are named integral membrane proteins [28]. Other proteins have one portion that is anchored in the membrane and extra-membrane regions that point into or out of the cell. The so-called peripheral membrane proteins remain firmly associated with the membrane surfaces [29]. The peripheral proteins interact with integral membrane proteins in important cellular processes such as energy metabolism and transport [29].

The main function of the cytoplasmic membrane is to prevent the passive leakage of solutes into or out of the cell; it acts as a gateway for the transport of nutrients into and wastes out of the cell (permeability barrier). The membrane is also an anchor for many proteins, glucides, lipids, and complexes of the same. Some are enzymes that catalyze bioenergetic reactions whereas other transport solutes into and out of the cell [26].

Salts, sugars, amino acids, nucleotides, and many other substances need to be transported by proteins that, besides ferrying substances across the membrane, accumulate solutes against the concentration gradient. One substance that can cross the membrane in both directions is water, but its movement is accelerated by dedicated transport proteins, called “aquaporins” [30].

#### 1.1.4. Pathogenic Bacteria

As previously mentioned, disease-causing bacteria are referred to as pathogens. However, in some cases, it is not possible to draw a clear distinction between pathogenic and non-pathogenic bacteria since the encounter of the human organism with the pathogen does not always generate disease. The severity and outcome depend on the complex relationship that is established between the microorganisms and the human organism. Under normal conditions, the thousands of bacteria that make up the microbiota are harmless to our health; indeed, they assist and help maintain it. The immune defenses of our body, therefore, help us to avoid the invasion of microbiota microorganisms, but when the body is compromised for various reasons, invasion can occur, and serious illness appears. Hence, the same beneficial bacteria can become pathogenic. Table 1 shows the different human pathogenic bacteria based on morphology and gram stain [24].

### 1.2. Bacterial Cell Wall Polysaccharides (CWPs) and Peptidoglycans (PGs): Structure and Functions

**Figure 2:**
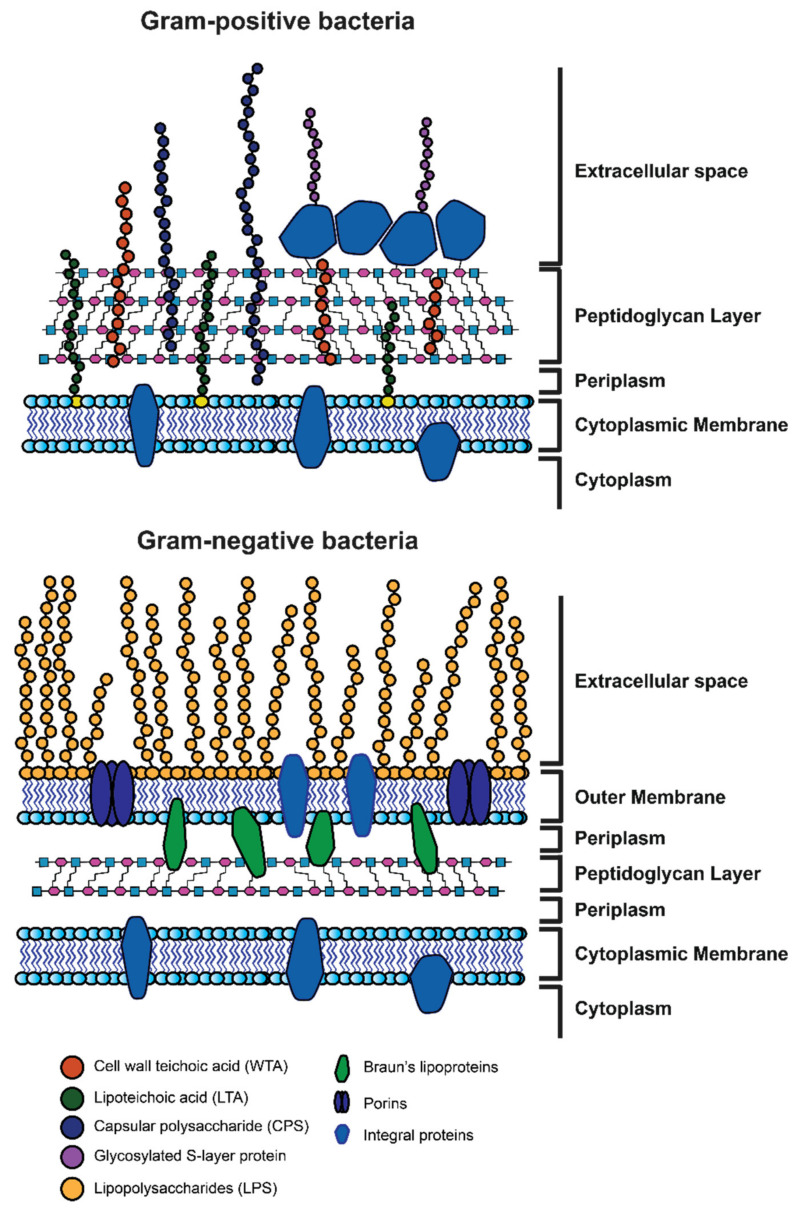
Schematic representation of the cell wall structure of gram-positive and gram-negative bacteria.

From a structural point of view, the bacterial cell wall can be classified into two different types, depending on gram-positive or gram-negative bacteria, as depicted in Figure 2. The main difference between the two cell walls is the absence of a second bilayer membrane in the gram-positive bacteria, which is compensated by a thicker peptidoglycan (PG) layer. The bilayer membrane in gram-negative bacteria is highly asymmetric; the inner leaflet is composed of phospholipids while the outer leaflet mainly contains lipopolysaccharides (LPS) whose polysaccharide chains occupy the extracellular space (Figure 2). [31,32] The PG layer in gram-positive bacteria is a fundamental component of the cell wall placed outside the cytoplasmic membrane of almost every bacterium [33,34,35], with very rare exceptions (e.g., *Mycoplasmas*, *Planctomyces*, *Orientia* (*Rickettsia*) *tsutsugamushi*) [36,37]. Its primary role is to maintain a sustained turgor of the cell wall, preserving the integrity and counteracting the osmotic pressure of the cell [38,39], but it also represents the anchor point for proteins and cell wall polysaccharides (e.g., teichoic acids) [40,41]. Together with the assembly of bacterial peptidoglycan hydrolases, the PG is also deeply involved in the process of cell division and cell growth [42]. Given its almost ubiquitous nature, higher eukaryotes evolved several PG recognition molecules (e.g., CD13, Nod1-2, lysozymes, and amidases) representing the first defense line against bacterial infections [38,43,44]. Moreover, PG represents a robust target for almost all effective antibiotics inhibiting bacterial cell wall synthesis [39]. The presence of such a layer is of fundamental importance for the integrity of the cell. Indeed, any degradation (e.g., by lysozymes) during cell growth or inhibition of its biosynthetic path leads irremediably to cell lysis. In addition to morphological functions, the cell wall undergoes recycling where it is constantly broken down, turned over, and remodeled [45,46,47].

PG is of major importance in gram-positive bacteria given its higher abundance which accounts for almost half of the cell wall mass, while in gram-negative bacteria, the PG layer is rather thin and not directly exposed to the extracellular space due to the presence of the outer membrane. Owing to its close to the omnipresent occurrence, PG has been one of the major antibiotic targets in recent years (e.g., penicillin, cephalosporins, carbapenems, vancomycin, teicoplanin) [48].

#### 1.2.1. Chemical Structure and Variability of PG and Correlated Functions

**Figure 3:**
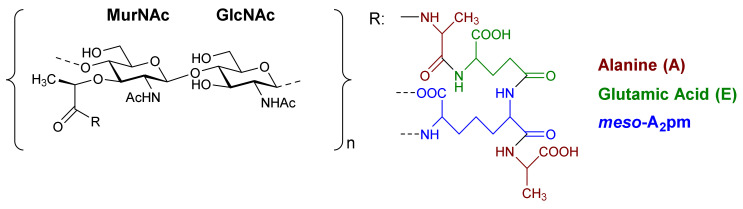
Key structural elements that are representative of the peptidoglycan region. The strands of PG are linked via peptide chains that condense with each other forming amide bonds via either the meso-A2pm carboxyl group or amine group.

The basic scaffold of the PG layer, as depicted in Figure 3, consists of polymeric glycan strands of two alternating β-(1→4)-linked sugars: *N*-acetylmuramic acid (MurNAc) and *N*-acetylglucosamine (GlcNAc). The different strands are arranged parallel to each other and covalently connected via peptide linkers that are substituted at the d-lactyl group of each MurNAc residue. The most common peptide linker is composed of l-Ala-γ-d-Glu-*meso*-A2pm (or l-Lys)-d-Ala-d-Ala. The last d-alanine residue is lost in the mature macromolecule and the previous d-Ala condenses with the corresponding d-Ala residue of another strand’s linker, thus creating the distinctive net of the PG layer.

Variations from the general structure of PG have largely been divided into three categories, *viz*., of: (i) the glycan strand structure and substitution pattern, (ii) the lipid linker structure and condensation point, and (iii) the glycosylation point by extracellular glycans. The most commonly reported modification in the first case is *N*-deacetylation and *O*-acetylation patterns at both GlcNAc and MurNAc, *N*-glycolylation of MurNAc residues, the modification of MurNAc to muramic *δ*-lactam, or formation of 1,6-anhydro MurNAc. The extent of these substitutions is species-dependent and has been extensively reported in previous literature works [49]. Table 2 shows the most common modifications of the PG repeating unit that have been discovered so far.

*N*-deacetylated sugars in the PG layer of *Bacillus* strains have been reported extensively for both residues [50,51,52], and more recently also for Streptococcal bacteria (e.g., *S. pneumoniae*, *S. pyogenes*, *S. iniae*) [53,54,55]. Deacetylation most likely occurs on the mature peptidoglycan since deacetylated precursors have not yet been reported in species containing PG deacetylases, and the latter enzymes have precise extra-cytoplasmic localization [54].

It is well known that *N*-deacetylation plays a key role in the protection of certain bacteria from host antimicrobial proteins such as lysozymes, which catalyzes the breakage of the β-(1→4)-linkage between GlcNAc and MurNAc leading to hydrolyzed PG layers and bacterial lysis [56]. This was shown to be related to the increased positive charge of the PG layer which decreased the efficiency of the secondary (non-enzymatic) mechanism of action of lysozymes [57], where these proteins cross the cell wall and bind to negatively charged bacterial membranes leading to its permeabilization. The lack of *N*-acetyl groups has been shown to provide a poor substrate for lysozyme binding, while upon chemical *N*-acetylation of the substrate lysozyme activity seems to be restored [54,58,59,60].

*N*-glycolylated muramic acids in PG, first described in *Mycobacterium smegmatis* [61], are used in bacterial taxonomy to classify *Actinomycetales*, a class of bacteria from which important antibiotics are isolated (e.g., streptomycin, actinomycin, streptothricin) [62]. Such a modification is widely present in the Mycolata taxon, a group of bacteria containing mycolic acids in their cell walls (e.g., *Mycobacterium, Rhodococcus*, *Tsukamurella*, *Gordonia*, *Nocardia*, *Skermania, Dietzia*) [63,64,65]. The modification is introduced during the synthesis of the UDP-precursors and generally is present only on a fraction of the muramic acid residues. Therefore, bacteria containing *N*-glycolylated muramic acids also contain MurNAc residues in the PG layer. The role of the *N*-glycoloyl group as a substituent has been the object of speculation but is still to be clarified. It has been proposed that the extra hydroxyl would engage in hydrogen bonding within the cell envelope with stabilizing effects [66].

Intramolecular amide bond formation between the amino group and the carbonyl of the lactyl group in MurNAc generates the muramic acid δ-lactam modification which appears to be quite abundant in *Bacillus* species and *Clostridium sporogenes* spores. For this modification to occur, the lactyl group must not have any peptide attached and the MurNAc residue must be *N*-deacetylated. This process is generally handled by just two enzymes. In *B. subtilis* for instance, an amidase CwlD removes the peptide that is linked to MurNAc and the peptidoglycan MurNAc *N*-deacetylase PdaA cleaves the acetyl group of the sugar residue [67]. Their role seems to be related to the spores’ ability to complete the germination process to produce viable cells. The muramic acid δ-lactam modification is employed by germination-specific hydrolases as a marker to recognize the spore’s PG [68,69,70].

The *O*-acetylation in PG appears to be uniquely present at the MurNAc residues [71,72]. First reported in *Micrococcus luteus* and *S. faecalis*, it is present in both gram-positive and gram-negative bacteria (e.g., *B. cereus*, *Staphylococcus aureus*, *Enterococcus hirae*, and *S. pneumoniae* for the gram-positive; *Neisseria gonorrhoeae*, *Neisseria meningitidis*, *H. pylori*, and *Proteus mirabilis* for the gram-negative ones). As a structural variation, *O*-acetylation is more prevalent than *N*-deacetylation and the extent of it varies between <20% and 70% depending on the species and strain that is being considered [73,74,75]. The source of acetyl groups for the *O*-acetylation has been a matter of discussion over the years. It has been proposed to be extracted from the N-2 position of GlcNAc or MurNAc [76]. But there are cases where acetylation in MurNAc6Ac is present in fully *N*-acetylated PG (e.g., *P. mirabilis*); alternatively, it was suggested to be obtained from peptidoglycan turnover products [73]. More recent theories have proposed acetyl-coenzyme A (CoA) or acetyl phosphate to be the source [77].

*O*-acetylation of PG has an important role in the ability of bacteria to produce disease. The first experimental evidence suggested this substitution to be responsible for the increased resistance towards hen egg-white lysozyme hydrolytic activity [72]. Further shreds of evidence showed the same resistance to egg white lysozyme on 14 strains of *P. mirabilis* containing acetylated PG, and further species lacking *O*-acetylation patterns were shown to be more sensitive to exogenous lysozymes [42,77,78]. Therefore, *O*-acetylation is a contributing factor offering resistance against antimicrobial proteins. Interestingly, the degree of *O*-acetylation seems to be negatively affected by β-lactam antibiotics that target proteins that are related to transpeptidation reactions in the synthesis of peptidoglycans. *O*-acetylation of peptidoglycan appears to play a not-yet resolved role in the cross-linking reaction [79,80,81].

#### 1.2.2. Cell Wall Polysaccharides: Structure and Functions

Gram-positive and gram-negative bacteria produce extracellular polysaccharides which can either be covalently bound to the cell envelope or surface proteins or be a slime that is weakly attached to the bacterial cell wall. Being present at the interface between microbes and mammalian hosts–other than representing recognition elements for pathogens–these glycoconjugates often contain antigenic moieties that trigger an immunogenic response in hosts. For this reason, these glycans and their biosynthetic pathways have experienced increased attention from the scientific community during the last decades.

**Figure 4:**
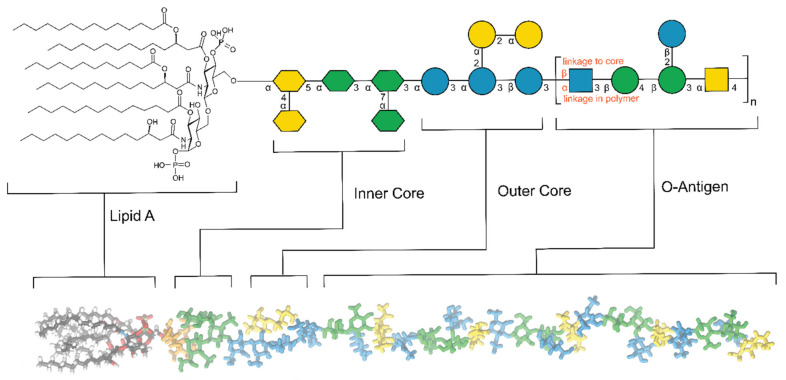
Lipopolysaccharide with its O-antigen from *E. coli* O6 built with *LPS modeler* [83] and rendered in VMD. Figure adapted from Furevi [84].

As a result of Red Queen evolutionary dynamics [82], the structures of bacterial glycans present the highest variability compared to other non-bacterial classes of glycans and cannot, therefore, be easily generalized. As previously reported, the term “Red Queen” effect takes inspiration from Lewis Carroll’s quote “it takes all the running you can do, to keep in the same place” borrowed to explain the glycan structural variation that contribute to the bacterial diversity in nature. As above mentioned, bacteria have historically been divided based on the outcome of the gram staining procedure. In gram-negative bacteria, glycans are mostly present in the form of lipopolysaccharides (LPSs). A typical structure of an LPS polymer is represented in Figure 4.

LPS is generally divided into three sections: the lipid A, the core oligosaccharide (inner and outer core), and the O-antigen polysaccharide. The lipid A (or endotoxin) is the innermost and hydrophobic region of the lipopolysaccharide and possesses a conserved structure that is composed of two β-(1→6)-linked glucosamine residues often phosphorylated at the reducing end and non-reducing end residues in positions 1 and 4′, respectively. Acyl groups are condensed at the hydroxyl and amine groups in a variable number [85,86]. Due to this common architecture, most of the lipid A moieties are detected at picomolar levels by receptors that are present on immune system macrophages and endothelial animal cells [86], and a multitude of enzymes that are linked to the lipid A biosynthesis (e.g., LpxC) have over the years been validated as a target for newly developed antibiotics.

Discovered in the late 1800s and isolated from *E. coli*, it was confirmed that LPS was responsible for the pathogenic (endotoxic) properties, which correlated to pathological outcomes (e.g., fever and septic shock). Lipid A is part of pathogen-associated molecular patterns (PAMPs), i.e., molecules that can activate innate immune systems when they are recognized by toll-like receptors (TLRs) or other pattern recognition receptors (PRRs).

Roughly 10^6^ lipid A residues are present in a single cell of *Escherichia coli* [87]. The fatty acids are somehow variable in structure and chain length but only one type of fatty acid is amide-linked, i.e., (*R*)-3-hydroxy fatty acids. The length of these fatty acids is 10–20 carbon atoms per chain and if the third carbon atom carries a hydroxyl group it is most often substituted by an additional fatty acid in at least one of the sugar residues [88,89,90,91].

Some species have been shown to contain a keto group in place of the hydroxyl in the *N*-acyl fatty acids [92,93]. Besides that, (*R*)-3-hydroxy fatty acids are predominant also for the ester-bound fatty acids, but these are still more variable and species-dependent. These are fatty acids from myristic and lauric acids and their hydroxylated derivatives, to even more unique structures such as *cis*-11-octadecenoic acid, 3-hydroxy-5-dodecenoic acid, and iso-2,3-dihydroxytetradecanoic acid [94].

The lipid A is covalently linked to heteropolysaccharides via a core region consisting of two parts, viz., first an inner core containing one to four units of Kdo (3-deoxy-d-manno-oct-2-ulosonic acid) [95], as well as l-*glycero*-d-*manno*-heptose residues, which are found in many inner cores. Additional charged elements such as phosphate and uronic acids may be present in the inner core structure providing a binding site for divalent cations (Mg^2+^ and Ca^2+^) with the effect of stabilizing the outer membrane [96].

The second portion of the core, referred to as the outer core is slightly more variable but structural modifications are still limited if compared to the O-antigen. This last complex repeating unit determines the serological and antigenic properties of the LPS and possesses a high structural variability. This diversity is reflected in the wide range of residues composing the repeating unit, which includes uronic acids, amino sugars, methylated and acetylated derivatives, deoxy sugars, but also non-sugar moieties such as amino acids and phosphate groups. The variability is further extended by the linkage diversity between the different residues in the branched and non-branched repeating units. The number of sugars in each repeating unit is typically four or five, although up to seven sugar residues have been reported for *E. coli* [97]. The total number of residues in a single chain can add up to several hundred, as for some bacterial species up to one hundred repeating units have been shown to be the outcome of the O-antigen biosynthesis. Hence, even a single bacterial species can account for hundreds of different O-antigen structures. *E. coli* is one of the most characterized species in terms of serotypes having–to date–197 reported O-antigens [97].

Over the years databases have been created to collect the different O-antigen structures of various bacterial species from a structural and genetic point of view [98,99,100]. Gram-positive bacteria contain a wide range of glycans on their cell wall. Similarly to glycans from gram-negative bacteria, these are important structural elements with significant patho/physiological relevance, other than representing key surface units for cellular recognition and signaling. Some of the most common and well-studied glycans from gram-positive bacteria are the cell wall teichoic acids (WTAs), which consist of copolymers of glycerol phosphate or ribitol phosphate and carbohydrates that are linked together by phosphodiester bridges.

These polyanionic glycopolymers play critical roles in the cell with functions that include cell morphology and division, autolytic activity, antibiotic resistance, metal ion homeostasis, phage-mediated horizontal gene transfer, and protection of bacteria from host defense peptides and antimicrobial peptides [101,102]. For their importance, these structures and their biosynthetic pathways represent attractive targets for the design of antibiotics and vaccines.

**Figure 5:**
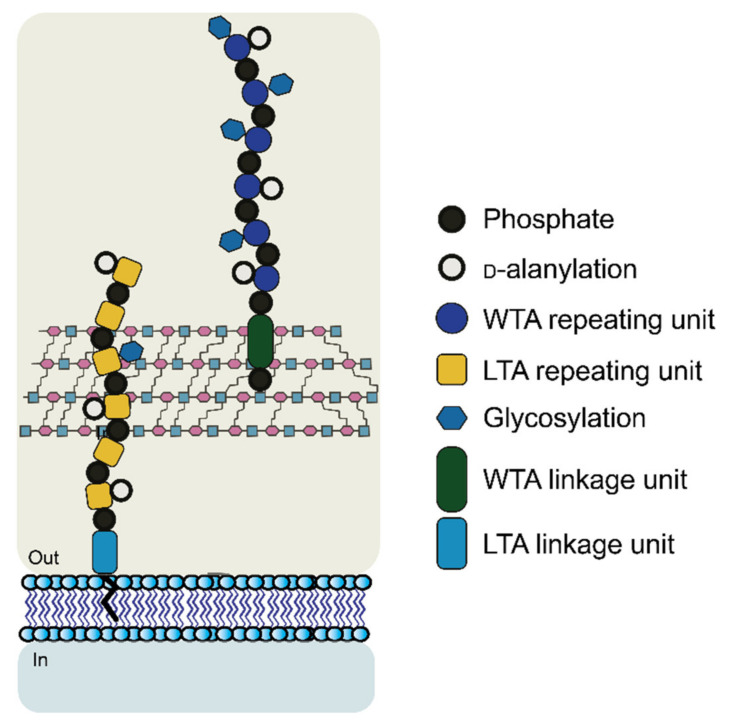
Schematic representation of LTA and WTA in the gram-positive cell wall. The WTA extends beyond the PG layer while LTA may not be able to become elongated past it.

The WTA polymers can account for up to 60% of the cell wall mass containing between 40 to 60 polyol repeats. Structurally, WTA can be divided into two parts: the main chain polymer and a linkage disaccharide that acts as a bridge between the main chain and the peptidoglycan (Figure 5) [101,103,104].

The disaccharide unit is highly conserved across species and its structure is β-d-ManNAc-(1→4)-α-d-GlcNAc-1-*P*, where *P* represents a phosphodiester group that is linked to the hydroxyl group at position 6 of *N*-acetyl muramic acid of the PG layer. The *N*-acetyl-β-d-mannosamine residue is substituted in position O4 with one to two glycerol 3-phosphate units [105,106].

The most common WTA repeating units contain ribitol 5-phosphate (RboP) or glycerol 3-phosphate (GroP), but there is great variability in the WTA monomer structure with more unusual moieties [107,108,109]. Additional structural diversity may arise from the presence of substituents that are attached to the hydroxyl groups of the polyol repeating units, e.g., d-alanine, monosaccharides such as Glc or GlcNAc, or oligosaccharides [110].

The presence of these substituents is important in bacterial defense against a host and antibiotics. The d-alanylation, for instance, has been proven to protect against host-defense mechanisms [111,112]. A reduction or complete absence of d-alanine residues was shown to be correlated with an increased susceptibility to phagocytes, neutrophils, lysostaphin, and lysozymes, together with glycopeptide antibiotics and cationic antimicrobial peptides [113,114,115,116,117]. These structural changes may thus be correlated to the overall change in the charge of the membrane [116]. While the specific removal of β-d-GlcNAc modification has been reported to increase sensitivity to β-lactam antibiotics [118].

The second most characterized cell-wall glycopolymer in gram-positive species are the lipoteichoic acids (LTAs), which, similar to WTA, are zwitterionic polymers, but with a simpler structure compared to WTA. LTA typically consists of a polyglycerolphosphate (PGP) chain that is linked to the bacterial membrane via a glycolipid anchor (see Figure 5) [119]. As in WTA, the backbone of LTA is modified with alanine or glycosyl residues. Their structures also contain an initial linkage unit which is mostly represented by a disaccharide that is linked to diacylglycerol (DAG). In *S. aureus* and *B. subtilis*, the disaccharide unit is *β*-d-Glc(1→6)-*β*-d-Glc.

LTA can coexist with WTA in the same bacteria and their functions are generally similar. In bacteria containing only LTA moieties, the lack of these may lead–in addition to the same roles that are observed for WTA–to morphological defects such as the increased size of the bacteria or temperature-sensitive growth phenotypes [120,121]. Besides their location in the membrane, where LTA are tethered to the membrane via van der Waals forces and WTA are covalently attached, the LTA generally do not exceed in length past the peptidoglycan membrane while WTA extends further out into the extracellular space.

While WTA and LTA structures are possibly the most common and well-known membrane glycans in gram-positive bacteria, the cell walls of many gram-positive bacteria of the genera *Streptococcus*, *Enterococcus* and *Lactococcus* lack these polyanionic structures and have evolved glycopolymers that are characterized by the presence of rhamnose. These rhamnose-containing CWPs (RhaCWPs) are of particular interest due to the total absence of l-rhamnose in humans, which makes their biosynthetic pathway an attractive therapeutic target. Indeed, l-rhamnose is very often essential for bacterial virulence or viability [122].

**Figure 6:**
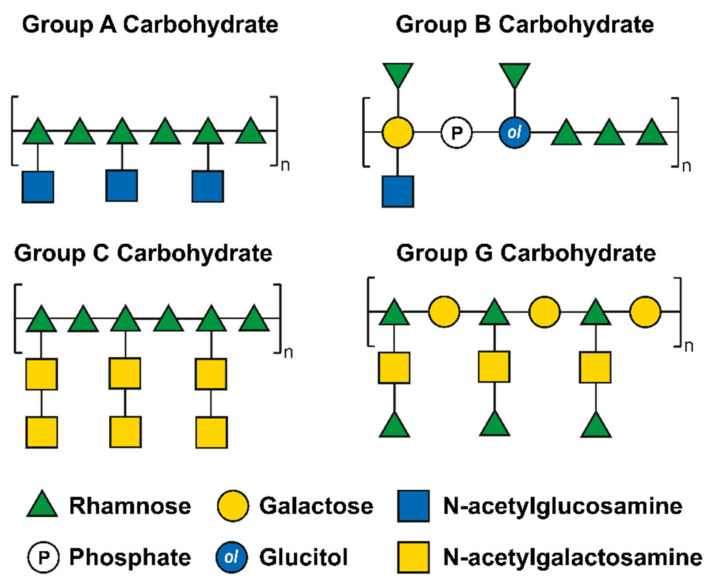
Different types of streptococcal glycan repeating units according to the Lancefield classification. The representation follows the shapes and colors of the SNFG representation for carbohydrates [125].

First reported by Rebecca Lancefield in the 1930s, these structures have historically been used in the serological classification of streptococci, which initially were used to define Group A–E streptococci and later expanded to more than 20 serogroups [123,124], Figure 6 [125].

Over the years, the classification became superfluous since it was found to be unable to differentiate between different species. It is still commonly accepted that a single streptococcal species can express different Group antigens (e.g., *Streptococcus dysgalactiae* subsp. *equisimilis* strains) [126]. Similarly, to WTA, the RhaCWP comprises up to 60% of the dry cell wall mass. They are mainly localized on the outermost surface of the cell wall, but they can also be found intercalated in the peptidoglycan net [127,128,129].

Streptococcal species seem to lack orthologues of the genes that are associated with the expression of WTA (e.g., TagB, TagD, TagF) [130], while Lactococcal and Enterococcal species were reported to contain orthologues for WTA and both WTA and LTA biosynthesis, respectively, thus having a more heterogeneous expression of glycans on their surface [131,132,133,134,135]. The main constituents of the RhaCWP are rhamnose with variable combinations of Glc, GlcNAc, Gal, GalNAc, and phosphate groups, that are differently linked [136]. Even though the structure of the polymers may vary significantly across species, some exceptions show structural similarities, such as GAC and GCC. These different motifs reflect the discriminatory ability of the Lancefield scheme that is based on the diverse structural elements that are present in the different groups. Some of the known structures for the Lancefield Groups A, B, C, and G are shown in Figure 6. Variations of these structures have been reported in different works and serve as homologs of WTA in terms of biological functions [137].

The first studies on the biological functions of RhaCWP indicated a strong relationship between these and the structural identity of the cell wall [138]. The lack of RhaCWP expression was shown to lead to overgrowth and cell division abnormalities [138,139,140,141]. Inhibition of the UDP-GlcNAc:lipid phosphate transferases in *S. galactiae* [140] and *S. pyogenes* [142] was shown to cause important morphological alterations such as a reduced level of cross-linked PG, increased chain length, and mislocalization of PG hydrolases.

RhaCWP also represents an important phage receptor in many species, as demonstrated in several studies, forming epitopes in conjunction with side-chains of the polymer (e.g., GlcNAc, Glc) [143,144,145]. Additional roles are associated with the impact on virulence of the bacteria, modulated by structural modification on the RhaCWP, still maintaining unaltered bacterial physiology [133,142,146] and cell wall morphology [142]. Furthermore, knowledge of the biosynthetic pathways of RhaCWP facilitates the identification of attractive targets for developing new antimicrobial agents with limited risk of side effects due to off-target actions, since humans completely lack l-rhamnose in their system [147,148,149].

## 2. Drugs Targeting CWPs and PGs

### 2.1. Antibiotics

Common therapeutic strategies focus on the direct inhibition of the biosynthesis of the cell wall polysaccharides, blocking bacterial growth and host infection. Some widespread and well-known drugs that are recognized as inhibitors of cell wall biosynthesis are *β-lactam drugs* (e.g., penicillin) and *glycopeptides* (e.g., vancomycin and teicoplanin). Other antibacterial compounds are known for a different mechanism of action, viz., by disrupting the membrane structures of bacteria, as carried out by, e.g., polymyxins and daptomycin [150,151,152].

### 2.2. Carbohydrate-Based Antibiotics Targeting CWPs and PGs: Glycopeptides and Lipoglycopeptides

Glycopeptides and lipoglycopeptides are the representative antibiotics that are known to target the synthetic pathway of the bacterial cell wall [153]. Glycopeptide antibiotics are very important in the antibacterial treatment of gram-positive and gram-negative bacteria. In particular, lipoglycopeptides can dimerize, enhancing the binding affinity (hence the overall potency) for the peptidoglycan cell wall. The membrane anchoring is mainly achieved by the hydrophobic moieties [154].

**Figure 7:**
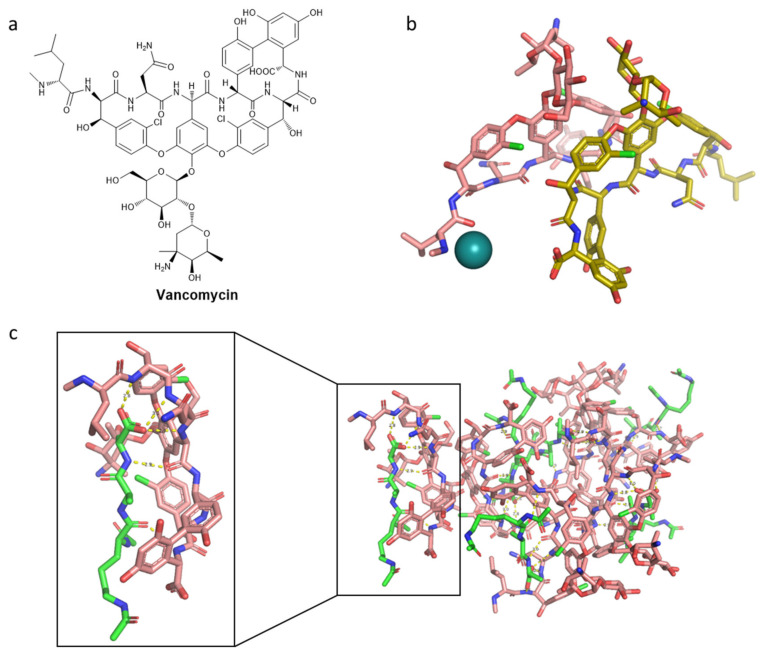
(**a**) 2D structure of vancomycin. (**b**) PyMOL 3D structure of two vancomycin molecules with a Zn^2+^ ion (PDB code 5M2K, ethanediol was removed); (**c**) 3D representation of the complex of vancomycin (colored in salmon) and d-Ala-d-Ala (in green, PDB ID: IFVM).

Vancomycin, depicted in Figure 7, is a glycopeptide drug, whose name comes from the word “vanquish”. In the 1950s, it was found in *Streptomyces orientalis* (now *Amycolaptosis*) resident in the Borneo jungle [155]. It was demonstrated in 1958 to be clinically important in the treatment of gram-positive penicillin-resistant staphylococci. Since then, it has been the best treatment for hospitalized patients with severe infections [153]. Due to its potential toxicity and the rise of semisynthetic penicillin that is potent on staphylococci, the interest in vancomycin decreased. However, some drug-resistant gram-positive bacteria became a threat, such as methicillin-resistant *Staphylococcus aureus* (MRSA); hence, vancomycin returned as an antibiotic. In routine treatments, vancomycin has been indicated for important infections such as meningitis, endocarditis, osteomyelitis, bacteremia, and skin infections that are caused by gram-positive strains. In particular, it is crucial in cases of intolerance-allergy to β-lactam antibiotics, or in the case of MRSA [153].

In the 1980s, vancomycin-resistant strains of gram-positive bacteria spread, for example, vancomycin-resistant enterococci (VRE), even if vancomycin is recognized to have a broad spectrum of activity [156]. Vancomycin showed its potency against almost every strain of *Staphylococcus aureus*, including the methicillin-resistant ones. *Leuconostoc* spp., *Listeria monocytogenes*, and *Lactobacillus* spp. are the ones that are not sensitive to vancomycin efficacy [153]. The broad spectrum of vancomycin includes most *S. epidermidis*, all *Streptococcus pneumoniae* [157], and all *Streptococcus pyogenes* that have been tested, most of *Streptococcus bovis* and *Streptococcus viridans*, and *Enterococcus* (only a few resistant) strains. Diphtheroids, the few strains of *Listeria monocytogenes*, most strains of *Clostridium species* (but some strains of *C. ramosum* are slightly resistant), half of the strains of *Actinomyces* species are also vancomycin-sensitive. All strains of the gram-negative bacillus *Flabacterium meningosepticum* resulted in resistance [158].

Considering the mechanism of action of vancomycin, it has a different mode of inhibition of cell wall biosynthesis in comparison to β-lactams. It prevents the polymerization of the phosphodisaccharide-pentapeptide-lipid complex, in the second biosynthetic step consisting of cross-linking between the peptide chains [159]. Vancomycin strongly binds to peptides presenting a d-alanyl-d-alanine repeating entity at the free carboxyl end [160]. Vancomycin is a bulky drug; hence, it creates an extensive steric hindrance when it is bound to the peptide, not allowing for binding to the substrate site on the enzyme peptidoglycan synthetase. Thus, it blocks the peptidoglycan biosynthesis. As its secondary mode of action, vancomycin was also more recently shown to interfere with the cell membrane permeability [161,162].

Vancomycin (Figure 7) is still essential in the treatment of methicillin-resistant staphylococci infections, with the help of other important antibiotics: first- and second-generation cephalosporins, co-trimoxazole, clindamycin, or rifampicin. However, as already mentioned, it is relatively toxic, with a high incidence of side effects (e.g., red man syndrome). It is also not intramuscularly administrable, it is expensive, and not so active against enterococci [163].

Another important glycopeptide is teicoplanin. It was isolated from the actinomycete *Actinoplanes teichomyceticus* [164], with the original name of teichomycin A2. Teicoplanins refer to five structurally similar glycopeptide antibiotics, possessing a tetracyclic backbone, which is reminiscent of the tricyclic motif of vancomycin. It is a reliable alternative to vancomycin, with different advantages in comparison to the latter due to its clinical efficacy at lower dosages in severe staphylococcal infections [163]. Other advantages are the lower incidence of side effects, such as the already-described red man syndrome and the lower toxicity (in particular in co-administration with an aminoglycoside) [165]. However, teicoplanin has been commercialized in Europe and Asia, but not in the USA [153].

Many types of gram-positive bacteria are sensitive to teicoplanin, which has bacteriostatic and bactericidal activity). Some families are involved in in vitro sensitivity, such as staphylococci (even the β-lactamase-producing and methicillin-resistant strains; *S. aureus*, *S. epidermidis*, *S. hominis*, *S. haemolyticus*, *S. saprophyticus*), streptococci (*S. pyogenes*, *S. pneumoniae*, *S. agalactiae*, *S. bovis*, *S. milleri*, *S. mitis*, *S. sanguis*, etc.), enterococci, and several anaerobic ones (*Clostridium difficile*, *Clostridium perfringens*, *Propionibacterium acnes*, *Listeria monocytogenes*, and *Corynebacterium jeikeium*).

**Figure 8:**
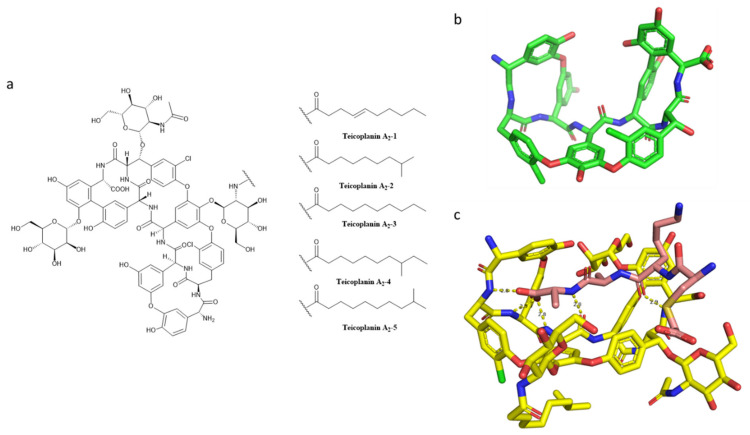
(**a**) 2D structure of teicoplanin, in its different forms. (**b**) 3D structure of teicoplanin aglycone (TAG: PDB ID 6TOV), DMS (dimethyl sulfoxide was removed from the original pdb file of the crystal structure) [171], and (**c**) teicoplanin in complex with d-Ala-d-Ala residue with polar contacts (PDB ID: 3VFJ).

Teicoplanin, depicted in Figure 8, shares the same mechanism of action with vancomycin: saturation by aspecific binding to the outer layers of PG, then binding to the terminal amino acyl-d-alanyl-d-alanine in PG. It follows a blockade of PG elongation, inhibiting the biosynthetic step of transglycosylation and interfering with the function of transpeptidases [166,167,168]. This glycopeptide has an “open pocket” conformation; the “seam” is represented by the heptapeptide backbone [169]. The unit *N*-acyl-d-alanyl-d-alanine fits into the pocket [170]. In particular, teicoplanin inhibits the biosynthesis of the cell wall and cellular growth. Most likely, cell death is induced by cellular hydrolytic enzymes. The PG of gram-negative bacteria is sensitive to teicoplanin, but the outer lipid membrane repels this bulky and polar drug [166,170].

In a comparison of teicoplanin to vancomycin, the first is several times more potent against enterococci compared to the latter. The relationship with other antibiotics could be different; the co-administration with rifampicin is additive or sub-additive, and the mix with ampicillin antagonizes the β-lactam-based antibiotic. However, teicoplanin has a certified, but also combination-dependent, synergic effect on gram-positive cocci with other antibiotics, such as aminoglycosides, imipenem, and fosfomycin.

**Figure 9:**
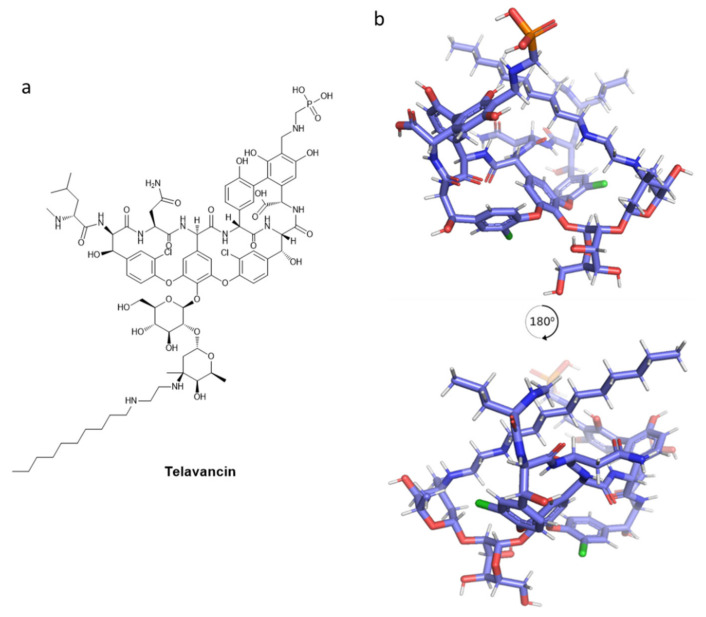
(**a**) Telavancin 2D structure and (**b**) 3D conformation of the minimized structure of telavancin (purple) that was obtained with PyMOL.

As above mentioned, vancomycin is crucial for antibacterial treatment, mainly for the worldwide spreading of MRSA [172,173]. Clinical failure is step by step increasing for MRSA treatment [174]; hence, it brought about the more effective semi-synthetic (lipo)glycopeptide antibiotics (with prolonged efficacy, thus less frequent dosage), such as telavancin, dalbavancin, and oritavancin [153,175]. A disadvantage is their accessibility as intravenous administration and their limited use because of their high cost. They mainly have a therapeutic indication for skin infections. The side effects are also a significant issue for these semi-synthetic lipopolysaccharide antibiotics: QT (the interval between the start of Q wave to the end of T wave measured on an electrocardiogram) prolongation and nephrotoxicity for telavancin and an increase of coagulation episodes for telavancin and oritavancin [153]. Telavancin (TD-6424, or Vibativ) is a vancomycin-derived lipoglycopeptide (for the decylaminoethyl substituent) which represents a valuable drug against the increased spreading of MRSA infections, and in general, infections that are caused by resistant gram-positive bacteria [175,176]. 2D and 3D structures of telavancin are presented in Figure 9. To potentiate the vancomycin antimicrobial activity, telavancin was developed in the context to improve the hydrophobicity at the disaccharide amine moiety. In this case, the hydrophobic portion is represented by a decylaminoethyl side chain on the vancosamine part and a phosphonomethyl aminomethyl functional group in position 4 on aminoacid 7 [177]. Telavancin offers different advantages, more related to specificity and pharmacodynamics properties. An increased affinity profile is offered by the glycopeptide moiety toward the d-Ala-d-Ala-containing peptidoglycan intermediates, and the negatively charged phosphonic acid moiety provides a more efficient urinary excretion [177,178]. The drug shows fewer problems after infusion and could be administered just once per day [176].

FDA-approved telavancin is used for complicated skin and soft tissue infections (cSSTIs), hospital-acquired bacterial pneumonia (HABP), and ventilator-associated bacterial pneumonia (VABP) that is caused by susceptible isolates of *S. aureus*, without current specific treatment. Different trials were conducted on telavancin for MRSA bacteremia. A randomized Phase II clinical trial (NCT00062647) confirmed that all the patients were cured [179]. It was also investigated for the treatment of *Staphylococcus aureus* bacteremia (SAB) [180] in a Phase III clinical trial, randomized, open-label, non-inferiority trial (NCT02208063), which failed due to insufficient statistical data [181,182]. More specific trials are needed to assess further the efficacy of telavancin, investigating the patients with complicated bacteremia more extensively [182]. Its mechanism of action, recognized to have dual activity, is mainly due to the interaction between the carboxylate binding pocket with terminal d-Ala-d-Ala residues and the decylaminoethyl side chain with the cell membrane [178,182]. Focusing on the d-Ala-d-Ala terminus, telavancin inhibits the peptidoglycan biosynthesis binding to *N*-acetyl-glucosamine-*N*-muramylpentapeptide of the peptidoglycan. The consequences of telavancin are the inhibition of different biosynthetic steps, such as the transglycosylation (PG polymerization) and the subsequent transpeptidation (generation of cross-links). In MRSA cells, telavancin is 10-fold more active than vancomycin in the inhibition of peptidoglycan synthesis [178]. The second mechanism of action is achieved by the decylaminoethyl side chain, which targets the cell membrane. This affects the cell membrane potential, through rapid concentration-dependent dissipation. The consequence is the generation of membrane pores [178], which leads to the extracellular leakage of adenosine triphosphate (ATP) and K^+^ ions. This second activity is specific for bacterial membranes, not for mammalian biomembranes.

Clinical and surveillance studies involving the antibiotic spectrum and activity of telavancin have been described [183,184,185]. In general, telavancin showed remarkable in vitro activity against skin and skin structure infections and pneumonia that is provoked by gram-positive bacteria (pneumonia could not be treated with daptomycin because it does not target lung tissue) [176,183]. Telavancin is particularly active against bacteria that are resistant to methicillin, daptomycin, and linezolid. It is also indicated for the treatment of vancomycin-intermediate *S. aureus* (VISA) and heterogeneous VISA, *Clostridium* species, including *Clostridium perfringens* and *Clostridium difficile*, *Peptococcus anaerobius*, and *Bacillus anthracis*. A synergic activity was demonstrated for the co-administration of telavancin with gentamycin and rifampicin [185,186,187]. To visualize telavancin, a structure representation was generated through energy minimization via Chem3D and then Avogadro (algorithm Steepest descent, force field MMFF94s), resulting in potential energy minimized 3D structure (Figure 9).

Oritavancin (previously LY333328, then KIMYRSA™, or ORBACTIV^®^) is a “long-acting” intravenously administered lipopolyglycopeptide that is approved by FDA for adult patients with acute bacterial skin and skin structure infections (ABSSSIs) that are caused by gram-positive bacteria. It is known for its antibiotic activity, efficacy, and safety. Other minor, but important advantages are a simpler preparation, and shorter infusion time (1 h) in a lower volume (250 mL) [188]. The initial novelty of oritavancin was its activity against vancomycin-resistant *Enterococcus faecalis* [189]. With its different hydrophobic and hydrophilic groups, it differs from vancomycin having an additional unsubstituted saccharide moiety and an aromatic lipophilic side chain [189]. It has a higher potency compared to vancomycin [190].

As for the mechanism of action, it acts by killing rapidly growing bacteria in a concentration-dependent way (unlike vancomycin) [154]. The inhibition of the peptidoglycan synthesis is achieved via different pathways: transglycosylation blockade, transpeptidation (cross-linking) inhibition, and membrane potential perturbation (depolarization and increased permeability) [189]. As for the spectrum of activity of oritavancin, it acts against gram-positive bacteria, remarkably against nosocomial infections that are caused by vancomycin-resistant enterococci (VRE) [191,192,193,194].

**Figure 10:**
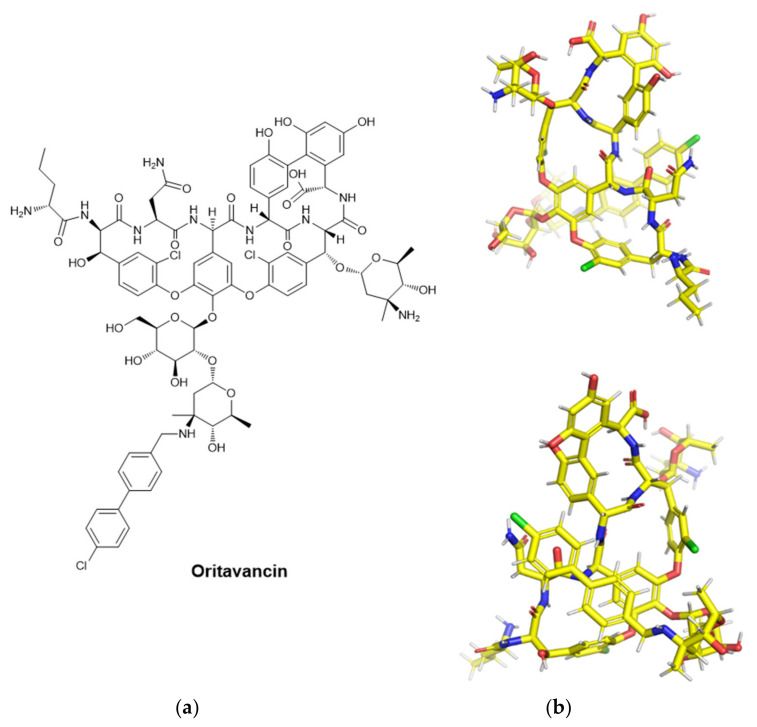
(**a**) 2D representation of oritavancin; (**b**) 3D energy minimized structure of oritavancin (yellow), made with PyMOL.

Other notable incidences were found against Micrococcus species, *Listeria monocytogenes*, and Corynebacterium species, but also anaerobic gram-positive bacteria such as *Clostridium difficile*, *Clostridium perfringens*, *Peptostreptococcus* species, *Peptococcus* species, and *Propionibacterium acnes* [191]. Oritavancin has a well-known synergy with gentamicin against VRE, and with gentamicin, linezolid, moxifloxacin, and rifampin [176,195]. There is no crystal structure involving oritavancin in the Protein Data Bank (PDB) [196]; hence, energy minimization via Chem3D and then Avogadro was performed, and the resulting conformation is displayed in Figure 10.

**Figure 11:**
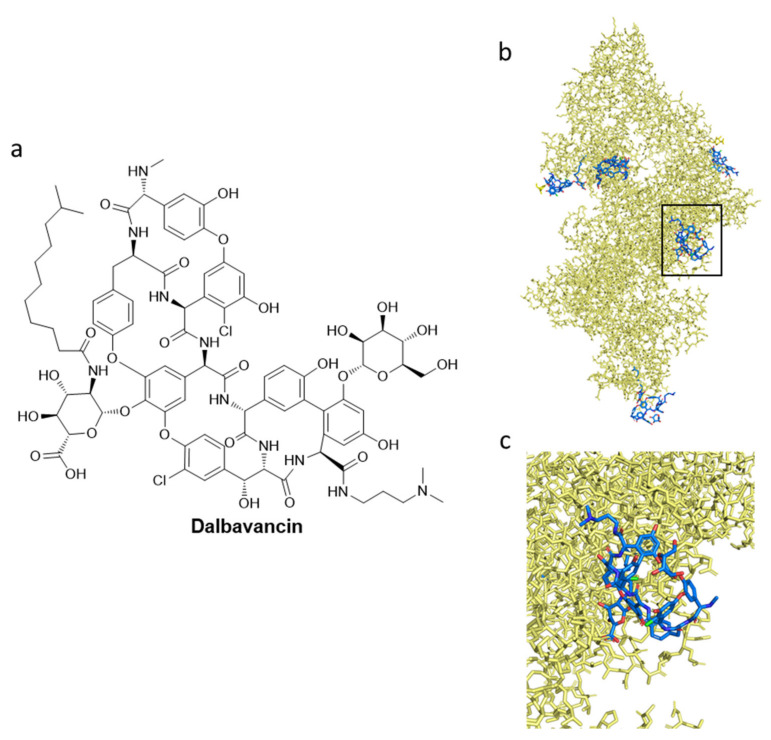
(**a**) Chemical structure of dalbavancin; (**b**,**c**) crystal structure (entire structure and close-up, respectively) of dalbavancin (blue) in complex with human serum albumin (HAS, yellow), PDB ID: 6M5E [198]. Figure made by ChemDraw and PyMOL.

The last semi-synthetic lipoglycopeptide that is presented is dalbavancin (Figure 11), which has a therapeutic indication for ABSSSI that is caused by gram-positive bacteria. In 2005, a Phase II clinical trial study demonstrated that dalbavancin is beneficial as an alternative to vancomycin for catheter-related bloodstream infections, even if more data are needed in support of this [197]. Dalbavancin has a good safety profile [192], but data are needed to evaluate its importance in MRSA bacteremia [182]. A Phase II clinical trial focused on the comparison between dalbavancin and the standard of care (SOC) therapy for the treatment of complicated bacteremia or right-sided endocarditis that is caused by *S. aureus* will be completed in August 2023 [182]. In Figure 11b,c, several structures of dalbavancin are presented as blue sticks, in complex with human serum albumin (HAS); their association with HAS is essential for clinical use [198].

### 2.3. Antibiotics Targeting CWPs and PGs: β-lactams, Polymyxins, and Daptomycin

**Figure 12:**
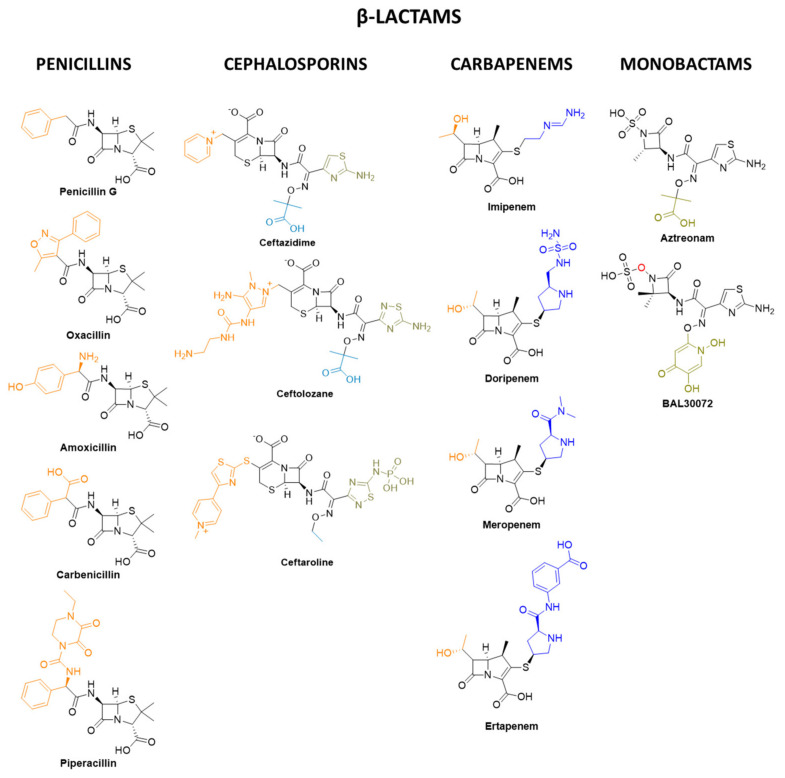
Structure of representative penicillins (penicillin G, oxacillin, amoxicillin, carbenicillin, and piperacillin); cephalosporins (ceftazidime, ceftolozane, and ceftaroline); carbapenems (imipenem, doripenem, meropenem, and ertapenem); and monobactams (aztreonam and BAL30072).

Due to the complexity of bacterial diseases (especially MDR ones), the treatment is carried out by combination therapies to gain activity through several mechanisms of action [199]. Penicillin, the “wonder drug”, is the oldest pure antibiotic worldwide that is available [200]. Penicillins (depicted in Figure 12) possess a β-lactam ring as the main chemical scaffold of the molecule.

The peptidoglycan (PG) layer generates cross-links at its C-terminus thanks to specific enzymes, transpeptidases (TPDs) [201]. The cross-linked PG polymer is essential for maintaining the membrane shape and prevents cellular osmotic rupture [150,151,201]. TPDs are PBP (penicillin-binding protein) enzymes because they can be inhibited by β-lactams such as penicillins [201]. β-lactams bind and inhibit PBP enzymes ending in bacteriolysis, hence cell death [150,151,201]. Penicillin-derived compounds are divided into natural (e.g., penicillin G), penicillinase-resistant (e.g., oxacillin), amino-, (e.g., amoxicillin), carboxy- (e.g., carbenicillin), and ureido- (e.g., piperacillin) penicillins. Natural penicillins are mostly active against gram-positive bacteria (streptococci, enterococci, and some non-β-lactamases-producing staphylococci), while synthetic ones (e.g., aminopenicillins) can be active also against some gram-negative bacteria such as non-resistant *H. influenza*, *N. gonorrhoeae*, and *E. coli*. Even if penicillin resistance is an increasing problem, this old class of antibiotics continues to lead to antibacterial therapy [202].

Moving forward into the family of β-lactam compounds, cephalosporins are important due to their activity against gram-negative bacilli. There are five generations of cephalosporins. Examples of cephalosporin-based therapies are available against most *Enterobacteriaceae* (third-generation ceftazidime + avibactam, their combined effect resulting in β-lactamase inhibition), resistant gram-negative bacilli such as *P. aeruginosa* (ceftolozane + tazobactam, another combination used for β-lactamase inhibition), and methicillin-resistant *S. aureus* (MRSA, with fifth generation ceftaroline as the only available treatment) [203].

Imipenem, doripenem, meropenem, and ertapenem (reported in Figure 12) are some of the drugs composing the well-known class of the carbapenems [203]. They derive from the naturally occurring antibiotic thienamycin, extracted by the soil microorganism *Streptomyces cattleya* [204,205]. They have the broadest spectrum activity compared to other β-lactam classes and are particularly important in case of severe sepsis that is caused by antibiotic-resistant bacteria. In general, they are active against both gram-positive (except methicillin-resistant *S. aureus*, *Enterococcus faecium*, and *Enterococcus faecalis*) and gram-negative bacteria as well as anaerobic species. [206]. The beneficial effect of carbapenems is reflected also in the significant post-antibiotic effect against gram-negative bacteria, such as *P. aeruginosa* [207,208]. Severe problems in using carbapenems for the treatment of infections are represented by the spread of carbapenemase-producing gram-negative bacilli [203].

Monobactams were developed in concomitance to the decrease of the efficacy of β-lactam antibiotics, mainly caused by extended-spectrum β-lactamases (ESBLs). These β-lactam-based drugs are structurally monocyclic and which makes them hydrolysis-stable compared to other β-lactam and are, therefore, interesting for new therapies [209,210]. Aztreonam (AZN, Figure 12) is currently the only available representative of this antibiotic family. It is only active against aerobic gram-negative bacilli [203]. Unfortunately, it is nephrotoxic and weakly immunogenic and is also very expensive. As with other β-lactams, aztreonam binds some transpeptidases and PBPs (only gram-negative ones), leading to cell lysis [211]. The potent activity against many gram-negative strains (also MDR) was demonstrated by BAL30072 (Figure 12), a derivative that entered Phase I clinical trials. However, BAL30072 has not progressed after Phase I in clinical trials in 2014 because of serious side effects, such as high alanine transaminase (ALT) levels in blood [212,213].

The class of lipopeptides is a broad-spectrum antibacterial-based family that is crucial to facing menaces of the highly-spreading MRSA [214] and the expansion of vancomycin-resistance MRSA infections [215,216,217,218,219,220,221].

Polymyxins are drugs of last resort but are still effective on dangerous bacteria such as carbapenem-resistant *Enterobacteriaceae*, MDR *Pseudomonas aeruginosa*, and *Acinetobacter baumannii* [216], also fosfomycin, ceftazidime/avibactam and meropenem–vaborbactam are an efficacious treatment for the above-mentioned bacteria [222,223]. They are known to disrupt the outer membrane of gram-negative bacteria [224], causing some antibiotic resistance problems [217]. Polymyxins [218,225] are non-ribosomal cyclic lipopeptides being secondary metabolites of a bacterium, *Paenibacillus polymyxa*; in particular, colistin (polymyxin E) [226,227], derives from *Bacillus polymyxa* var. *colistinus*. Structurally, they are decapeptides, consisting of a cyclic heptapeptide that is linked to a linear tripeptide side-chain acylated at the N-terminus by a fatty acid tail [228]. As for their mechanism of action, the primary electrostatic interaction with lipid A disrupts the outer membrane, following hydrophobic insertion of the fatty acyl chain of polymyxin into the membrane, making the bacterium succumb [229]. Attempts to extend the ring structure, generated by the insertion of additional Dab residues, produced compounds that are significantly less effective than polymyxins [27].

**Figure 13:**
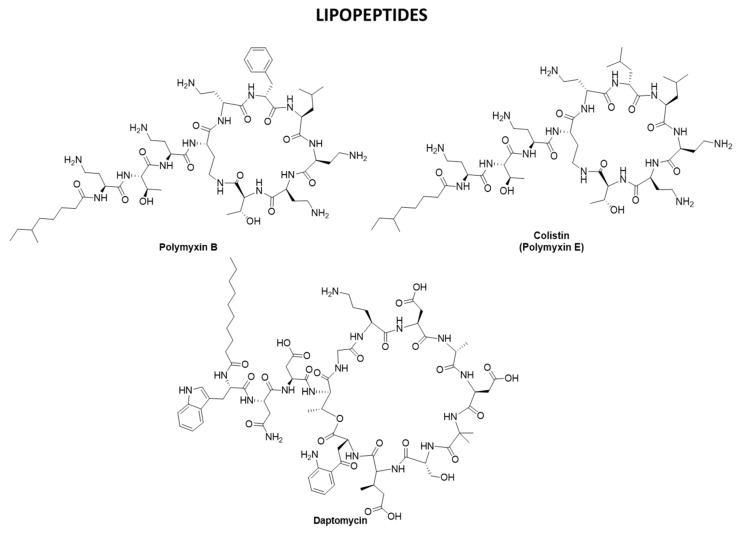
Structure of polymyxin B, colistin (polymyxin E), and daptomycin.

Daptomycin (Cubicin^®^), derived from *Streptomyces roseosporus* [229,230] has an in vitro selectivity against many MDR and susceptible gram-positive bacteria [231], including methicillin-sensitive and methicillin-resistant *S. aureus* (MRSA), streptococci, and *Enterococcus* (both vancomycin-susceptible strains and VRE) [153]. Daptomycin is not effective against gram-negative bacteria. In Europe, it is administered to adults intravenously once per day for cSSTI and complicated skin and skin structure infections whereas in the US it is used for the treatment of *S. aureus* bacteremia with or without right-sided infective endocarditis [153]. Its unique mechanism of action is extracellular and engages an oligomerization that is calcium-dependent [224]. Then, it binds to and alters the outer membrane, with a subsequent cellular potassium efflux. The final consequence is the depolarization of the membrane [153]. Figure 13 reports the 2D structure of polymyxins B, E (colistin), and daptomycin.

## 3. Antibiotic Drug Resistance

### 3.1. Consumption and Therapy

Since the discovery of penicillin in 1928, different classes of antibacterial drugs have been developed; unfortunately, bacteria mutate into resistant strains overcoming the mechanism of action of a specific drug. The increasing use of antibacterial agents made them gradually become less potent, also decreasing the therapeutic options that are available for patients that are affected by resistant strains. In the beginning, the problem was solved through the identification of new classes and the modification of existing antibiotics with limited cross-resistance to already in-use drugs. From the 1980s, the major scientific challenges in antibiotic discovery programs pushed large pharmaceutical companies to abandon this research area. Later, antibacterial resistance slowly became one of the main concerns in global clinics, pushing the scientific community, including some pharmaceutical companies, to re-invest money and efforts in the field. The main strategy that was pursued was to improve the potency of known antibiotic treatments, but new approaches to antibacterial drug discovery were also applied [232].

Recently, the World Health Organization (WHO) initiated a drug antibacterial drug development mapping activity and published a global clinical antibacterial pipeline report. The study highlighted that the clinical pipeline is dominated by derivatives of established classes and most development candidates display limited innovation, suggesting that the finding of new antibacterial agents without pre-existing cross-resistance should be prioritized in public funding strategies [233].

Regarding the preclinical phase, a review that was published in 2020 focusing on discovery and preclinical development projects has found, as of 1 May 2019, 407 antibacterial projects that were carried out from 314 different institutions, most of which are small and medium-sized enterprises (SMEs). The study evidenced that the preclinical pipeline contains 135 projects focusing on direct-acting small molecules belonging to innovative classes, directed towards new targets, or possessing new mechanisms of action, with high levels of diversity, and remarkable scientific concepts [234].

**Figure 14:**
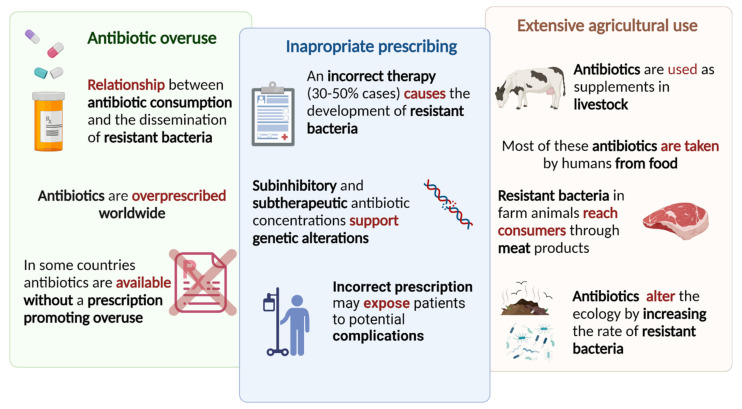
Summary of the roots of the antibiotic resistance crisis. Created with BioRender.com (Accessed on 29 April 2022).

Together with the scarce availability of innovative drugs, the antibiotic resistance crisis has its roots in overuse; epidemiological evidence indicates a direct relationship between antibiotic consumption and the emergence and dissemination of resistant bacteria strains. Despite the warnings, antibiotics are overprescribed worldwide, and, in some countries, antibiotics are unregulated, cheap, and available without a prescription, promoting their overuse. Inappropriate prescribing play a major role in antibiotic resistance and it has been estimated that antibiotic therapy (treatment indication, choice of agent, or duration of the treatment) is incorrect in 30% to 50% of the cases, strongly contributing to the development of resistant bacteria. For instance, sub-inhibitory and sub-therapeutic antibiotic concentrations support genetic alterations, such as changes in gene expression, horizontal gene transfer (HGT), and mutagenesis. Besides promoting the development of antibiotic resistance, the incorrect prescription may also expose patients to potential complications of antibiotic therapy. The extensive agricultural use of antibiotic agents as supplements in livestock to promote growth and to prevent infection were discovered to cause an ecosystem alteration that was related to the rising of resistant bacteria. Moreover, via the handling or consuming food, antibiotics reach consumers as well as resistant bacteria that developed in farms. A schematic representation of the main causes of the antibiotic resistance crisis is depicted in Figure 14. Most of these antibiotics are taken by humans from food; moreover, resistant bacteria in farm animals reach consumers through meat products. In addition, antibiotics that are used in livestock are excreted through urine and stool, and, together with sprayed antibiotics as pesticides, pollute the environment and alter the ecology by increasing the rate of resistant versus susceptible microorganisms [235].

An emerging tool, too long underestimated, that is useful to reduce the development of drug resistance is the prevention of bacterial infections and bacterial spread. Prevention can be pursued by developing new vaccines (vide infra Section 3.3) [236,237]. The prophylactic use of vaccines by reducing the diffusion of pathogens lowers antibiotic use and reduces multidrug resistance. This new role of vaccines in fighting drug resistance has been validate by several studies [238,239].

### 3.2. Drug Resistance Involving the Outer Membrane (OM)

Antibiotic resistance consists of a decreased sensitivity for a specific antibiotic, which allows the survival of the bacterium. Resistance to antibiotics is a natural phenomenon that has been noted since the introduction of penicillin in 1940 [240]. Very soon thereafter, the key question was raised, i.e., whether bacteria are inherently resistant to drugs or become resistant as a result of encountering the ‘antibiotic’ compounds. In search of an answer, it has been shown that bacteria can mutually transfer drug resistance to each other by passing genetic material between species. This process mainly occurs through the horizontal transfer of plasmids or bacteriophages, also known as horizontal gene transfer (HGT). Bacteriophages, i.e., viruses that are able to infect bacteria only, are also able to directly affect the bacterial genome. Moreover, the adaptation and evolution of microorganisms should also be taken into account in describing an integral view of the evolution of drug resistance [241].

**Figure 15:**
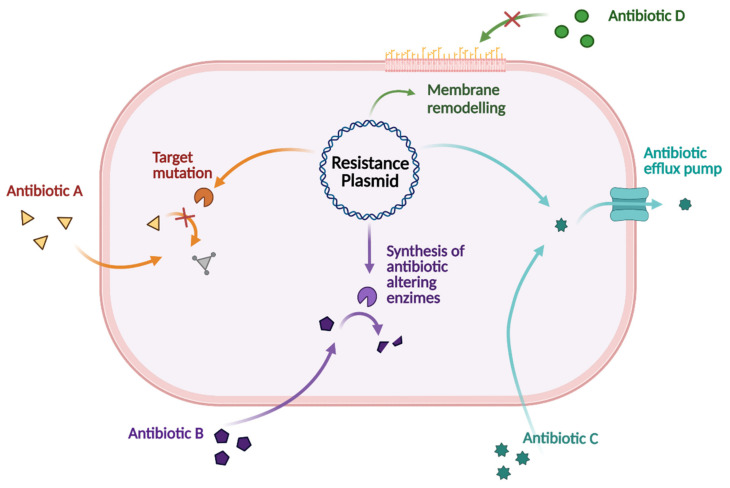
Scheme of mechanisms that lead phenotypic resistance evolution in bacteria. The highlighted paths include the mutation of the target (orange), the synthesis of antibiotic-altering enzymes (purple), the synthesis of genes encoding drug efflux pumps (light blue), and the membrane remodeling (green). Created with BioRender.com (Accessed on 29 April 2022).

There are four broad mechanisms that are indicated to lead to phenotypic resistance evolution in bacteria. A schematic representation of the bacterial mechanisms that lead to the evolution of phenotypic resistance is shown in Figure 15. The first one consists of the mutation of the target, resulting in the drug-binding inhibition. Multiple bacterial processes have been exploited as antimicrobial targets such as cell wall synthesis, nucleic acid synthesis, and metabolic pathways. All these targets could be modified by the bacteria to become resistant to the corresponding drug. A second option is related to the alteration of existing genes or the acquisition of new genes encoding drug efflux pumps. These efflux pumps extrude different classes of toxic agents, including a wide range of antibiotics, from the intracellular to the extracellular environment and their upregulation represents a crucial mechanism for acquired bacterial resistance. Also, the acquisition of new genes encoding enzymes that are able to modify or hydrolyze the specific drug could lead to a resistant phenotype. This process sequentially led to the acquisition of genes encoding for penicillinases, cephalosporinases, extended-spectrum β-lactamases, and carbapenemases. These enzymes are responsible for the decreased susceptibility of the related antibiotic class, the last of which has currently no solution. Lastly, the remodeling of the membrane could prevent drug influx at the cell surface, thus impeding access to the target that is located in the internal compartments [242,243,244].

Most antibiotics that are used in the 20th century bind specific targets that are exposed in the internal compartments of the bacterial cell, thus altering bacterial survival processes.

**Figure 16:**
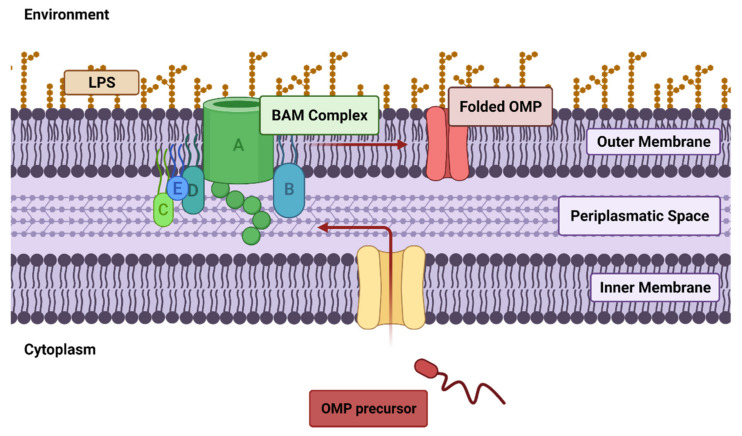
Biogenesis of bacterial OMP. Newly synthesized OMP precursors (red) move from the cytoplasm across the inner membrane and the periplasmic space. The β-barrel assembly machinery (BAM) complex (green), located in the OM, integrates the OMP precursors, creating the folded OMP (orange). Created with BioRender.com (Accessed on 29 April 2022).

In gram-negative species, the impermeable LPS-bacterial outer membrane (OM) strongly limits the access into the bacterium; entry of nutrients and other water-soluble molecules, drugs included, is controlled through the “major” (i.e., the most present) porins, aqueous pore that is abundantly expressed and largely integrated into the OM. Porins present a central luminal space that serves as an aqueous pore, which selectively allows the passage of hydrophilic molecules/drugs and whose expression is finely regulated [245]. For instance, in *E. coli*, the two genes OmpC and OmpF encode for the major porins; they are structurally almost identical, but their expression is modulated based on environmental conditions, such as hypo/hyperosmotic conditions (Figure 16).

In general, the OM proteome is frequently subjected to remodeling through two main pathways: the degradation of the present membrane components and the insertion of new proteins. Of the two biochemical processes, the latter has been quite unraveled; newly synthesized porin precursors move from the cytoplasm, across the inner membrane and the periplasmic space before arriving at the OM, where the β-barrel assembly machinery (BAM) complex integrates the porin precursors. Moreover, porin production is regulated in response to environmental changes by a complex system of regulators at both transcriptional and post-transcriptional levels. These regulators include many factors, such as small noncoding RNAs (e.g., micF, micC, micA), or two-component signaling systems, such as OmpR and CpxR [242].

Therefore, OM makes gram-negative bacteria, which are considered urgent pathogens by WHO, intrinsically less sensitive to a wide range of antibiotics compared to their gram-positive counterparts. For instance, gram-negative bacteria show natural resistance to vancomycin and teicoplanin. These two big tricyclic glycopeptides (M_W_ ≈ 1.45 and 1.7 kDa, respectively) interact with the terminal d-alanyl-d-alanine sequence on the pentapeptide side-chains of the peptidoglycan precursor, hence inhibiting peptidoglycan synthesis. Porins in the OM of gram-negative bacteria selectively allow hydrophilic molecules with M_W_ < 600 Da, thus impeding vancomycin and teicoplanin to reach their target. Therefore, they are considered gram-positive selective antibiotics [246].

Reduction in the number of porins and/or mutations modulating the selectivity of the porin channel could also contribute to limiting drug uptake. For instance, carbapenem resistance in some strains of *Enterobacteriaceae* was aimed at the reduction or abrogation of certain porins production. Conversely, imipenem and cephalosporins resistance in *E. aerogenes* and resistance to β-lactams and tetracycline in *Neisseria gonorrhoeae* were related to a modification in porins selectivity [243].

Since antibiotic resistance frequently results in a complex phenotype, the specific targeting of membrane remodeling, i.e., the alteration of membrane components (proteins or lipids) to allow bacterial adaptation to a new environmental condition, could offer a valid strategy to restore bacterial sensitivity to the available antibiotics. For instance, both the BAM and the transcriptional/post-transcriptional regulators could serve as a drug target, offering an innovative strategy to target OM remodeling.

Notably, OM has been identified as a potential target for nanoparticles that are designed for this purpose. For instance, it was recently reported that carbohydrate-coated silica nanoparticles were used to target *E. coli*. The study showed that gluconamide moieties on a nanoparticle coating interact with LPS molecules in the OM, allowing for penetration into the membrane without causing cytotoxic effects, thereby enabling drug delivery in a controlled way [247].

### 3.3. Carbohydrate-Based Antibacterial Vaccines

Vaccination is one of the key achievements in the fight against infectious diseases that has improved the length and the quality of human lives. It is the most efficient and least expensive strategy to prevent infectious diseases. It is based on the principle that stimulating an antigen-specific immune response provides protection from the respective eventual infection. The induced immune response includes the production of specific antibodies with long life and the formation of long-lasting memory B- and T-cells and it will be activated upon encounter with the pathogen [248].

Although vaccines were discovered and evolved from 1796 and onward it was only in the 1920s that the carbohydrate-based vaccines were developed. Avery and Heidelberger discovered that capsular polysaccharides (CPSs) from *Streptococcus pneumoniae* are immunoreactive components of the pathogen. This initiated the use of CPSs as antigens in the development of carbohydrate-based vaccines. Microbial pathogens carry a variously composed pattern of polysaccharides on their surface, based on oligosaccharide repeating units, in part functioning to evade the host immune system.

CPS antigens were discovered to give an age-dependent immune response and to be thymus-independent antigens and thus do not activate T-cells. The sole B-cell activation results in the production of low-affinity antibodies [249]. Protein-conjugated polysaccharides, zwitterionic polysaccharides and glycolipids, being thymus-dependent antigens, are capable of stimulating both B- and T-cells thereby generating high-affinity antibodies and immunological memory. The first CPS-based vaccine was a six-valent vaccine that was developed to prevent *S. pneumoniae* and was approved in 1947 but it was only in the 1970–80s that the more efficient CPS-conjugate vaccines were developed and approved [250,251]. The immunogenicity and efficacy of carbohydrate-based vaccines were effectively enhanced by coupling CPSs to carrier proteins. The proteins that were generally selected as carriers are denatured bacterial toxoids.

At present, several glycoconjugate vaccines are licensed and are commonly used to prevent *Haemophilus influenzae* type b, *Neisseria meningitidis*, *Streptococcus pneumoniae*, and *Salmonella typhi* [252]; a selection of FDA-approved vaccines are listed in Table 3, https://www.fda.gov/vaccines-blood-biologics/vaccines (Accessed on 29 April 2022).

The zwitterionic polysaccharides (ZPSs) contain both positive and negative charges in their repeating units. ZPSs activate the T-cells via electrostatic interactions, but only a small number of bacteria express zwitterionic polysaccharides on their surface. Therefore, chemical modification of a polysaccharide to incorporate also positively charged functional groups was performed to obtain a ZPS [253,254].

Glycolipids were discovered to have an immunomodulatory activity that is capable of activating a singular subset of T-lymphocytes, also known as invariant natural killer T (iNKT)-cells [255,256]. The immune potentiating properties of iNKT cells are currently being explored for the development of potential vaccines against metastasis, autoimmune, and infectious diseases [257,258]. The successful development of CPS-based vaccines first and glycoconjugate vaccines later stimulated the research on preventing further diseases such as cancer or infections that are caused by viruses, fungi, protozoan parasites, helminths, or other bacteria using carbohydrate-based vaccines [259,260,261].

## 4. Conclusions

Bacterial drug resistance is a worldwide threat to human health and both gram-positive and gram-negative bacteria can bypass host defenses to avoid the chemotherapeutic treatments of infection. The bacterial cell membrane functions as a barrier to the surroundings and whereas gram-positive bacteria have only a single cell membrane that is surrounded by a thick layer of peptidoglycan. Those that are defined as gram-negative contain two membranes, with only a thin peptidoglycan layer in between, and lipopolysaccharides in the outer leaflet of the outer membrane. Cell wall polysaccharides such as lipoteichoic acids and wall teichoic acids further complement the architecture of gram-positive bacteria. Therapeutic strategies to combat bacterial infection rely on the inhibition of cell wall biosynthesis employing β-lactam drugs such as penicillins or glycopeptides such as vancomycin and teicoplanin, whereas other approaches function by disrupting the membrane of bacteria, a mode of action that is used by polymyxins and daptomycin. Carbohydrate-based drugs that are used to treat infection that are caused by gram-positive bacteria include vancomycin, which blocks peptidoglycan biosynthesis. However, as vancomycin-resistant enterococci have emerged, treatment of these using existing antimicrobial agents poses significant difficulties. Furthermore, although drugs that are utilized in targeting gram-positive bacteria have been used in strategies for combating infections from gram-negative bacteria, by employing a combined use of rifampicin and colistin in combination with β-lactam drugs, the challenge of restraining and battling antibiotic resistance in gram-negative bacteria is paramount in future research.

## Figures and Tables

**Table 1:** Pathogenic bacteria and their associated infections. Examples of gram-negative and gram-positive pathogenic bacteria and their associated infections.

GRAM NEGATIVE	ILLNESSES
*Cocci*	
*Moraxella catarrhalis*	Respiratory tract infections
*Neisseria gonorrhoeae*	Gonorrhea
*Neisseria meningitidis*	Meningococcal Meningitis
*Chlamydia trachomatis*	Chlamydia
*Cocci-bacilli*	
*Rickettsia prowazekii* *Rickettsia typhi*	Epidemic typhus Murine typhus
*Cocci-bacilli and bacilli*	
*Brucella*	Brucellosis
*Bordetella*	Whooping cough
*Legionella*	Pneumonia
*Bacilli*	
*Escherichia coli*	Gastroenteric and urinary tract infections
*Salmonella typhi*	Typhoid fever
*Salmonella* spp.	Diarrhea
*Haemophilus influenzae*	Otitis, bronchitis, pneumonia, meningitis
*Flagellated bacilli*	
*Helicobacter pylori*	Stomach and duodenal ulcers
*Vibrions*	
*Vibrio cholerae*	Cholera
*Spirochetes*	
*Borrelia burgdorferi* *Leptospira*	Lyme disease Leptospirosis
*Non-fermenting Bacilli*	
*Pseudomonas aeruginosa*	Lungs, blood or heart valves infections
GRAM POSITIVE	ILLNESSES
*Cocci*	
*Staphylococcus aureus*, *S. epidermis*	Skin, heart valve and bone infections, pneumonia
*Enterococcus faecalis*, *E. faecium*	Hospital Intestinal infections
*Streptococcus B: S. agalactiae*	Infections of various systems
*Streptococcus A: S. pyogenes*	Necrotizing fasciitis, Acute rheumatic fever, Acute glomerulus nephritis, Scarlet fever
*Streptococcus pneumoniae*	Pneumococcal infections
*Bacilli*	
*Clostridium perfrigens*	Food poisoning
*Clostridium tetani*	Tetanus
*Clostridium botulinum*	Botulism
*Corynebacterium diphtheriae* *Corynebacterium jeikeium*	Diphtheria Severe infections in the hospitalized patient
*Actinomyces*	Actinomycosis
*Bacillus anthracis*	Anthrax
*Listeria monocytogenes*	Listeriosis
*Acid-fast bacilli*	
*Mycobacterium avium complex*	Lung infections
*Mycobacterium tuberculosis*	Tuberculosis
*Mycobacterium Leprae*	Leprosy
*Nocardia* spp.	Nocardiosis

**Table 2:** Known modification of the PG repeating unit.

Repeating Unit	Modifications
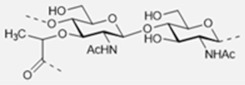	Unmodified unit
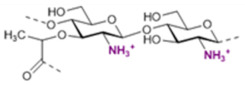	*N*-Deacetylation
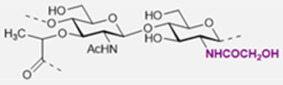	Glycolylation
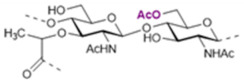	O-Acetylation
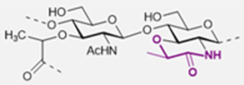	MurN-δ-lactam
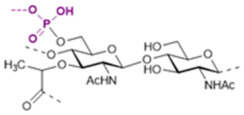	MurNAc-6-phosphate
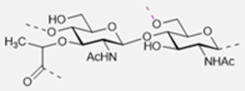	Linkage at GlcNAc

**Table 3:** Approved carbohydrate-based vaccines; a selection from FDA-approved vaccines. https://www.fda.gov/vaccines-blood-biologics/vaccines (Accessed on 29 April 2022).

Pathogen	Vaccine	Manufacturer and Trade Name
*Haemophilus influenzae type b (Hib)*	Glycoconjugate, polysaccharide with tetanus toxoid (TT)	Sanofi Pasteur (ActHIB^®^); GlaxoSmithKline Biologicals (Hiberix^®^)
	Diphtheria toxoid (DT), TT and acellular pertussis adsorbed, inactivated poliovirus and Hib–TT conjugate vaccine	Sanofi Pasteur (Pentacel^®^)
	Hib conjugate (meningococcal protein conjugate)	Merck & Co (PedvaxHIB^®^)
	Hib conjugate (meningococcal protein conjugate) and hepatitis B (recombinant) vaccine	Merck & Co (Comvax^®^)
*Neisseria meningitidis* *A, C, Y and W-135*	Glycoconjugate, meningococcal polysaccharide with DT	Sanofi Pasteur (Menactra^®^)
	Meningococcal polysaccharide	Sanofi Pasteur (Menomune-A/C/Y/W-135®)
	De-O-acetylated polysaccharide (C11 strain of MenC)	Pfizer (NeisVac-C^®^)
	Capsular oligosaccharide (C11 strain of MenC)	GSK (Menjugate^®^)
*Salmonella typhi*	Vi capsular polysaccharide	Sanofi Pasteur (TYPHIm Vi^®^)
*Streptococcus pneumoniae* *4, 6B, 9V, 14, 18C, 19F and 23F*	Pneumococcal polysaccharide 7-valent–CRm197 conjugate	Wyeth Pharmaceuticals (Prevnar^®^)
*Streptococcus pneumoniae* *1, 2, 3, 4, 5, 6B, 7F, 8, 9N, 9V, 10A, 11A, 12F, 14, 15B, 17F, 18C,19F, 19A, 20, 22F, 23F and 33F*	Pneumococcal polysaccharide, 23-valent	Merck & Co (Pneumovax 23^®^)

## Data Availability

Data sharing not applicable.

## Abbreviations

Outer membrane (OM); inner membrane (IM); peptidoglycan (PG); cell wall polysaccharides (CWPs); *N*-acetylmuramic acid (MurNAc); *N*-acetylglucosamine (GlcNAc); lipopolysaccharide (LPS); pathogen-associated molecular patterns (PAMPs); toll-like receptors (TLRs); pattern recognition receptors (PRRs); cell wall teichoic acid (WTA); ribitol 5-phosphate (RboP); glycerol 3-phosphate (GroP); lipoteichoic acids (LTAs); polyglycerolphosphate (PGP); diacylglycerol (DAG); rhamnose-containing CWPs (RhaCWPs); multidrug-resistant (MDR); methicillin-resistant *Staphylococcus aureus* (MRSA); vancomycin-resistant enterococci (VRE); complicated skin and soft tissue infections (cSSTIs); hospital-acquired bacterial pneumonia (HABP); ventilator-associated bacterial pneumonia (VABP); *Staphylococcus aureus* bacteremia (SAB); vancomycin intermediate *S. aureus* (VISA); acute bacterial skin and skin structure infections (ABSSSIs); horizontal gene transfer (HGT); Protein Data Bank (PDB); standard of care (SOC); human serum albumin (HAS); transpeptidases (TPDs); penicillin-binding protein (PBP); extended-spectrum β-lactamases (ESBLs); alanine transaminase (ALT); capsular polysaccharide (CPS); zwitterionic polysaccharide (ZPS); invariant natural killer T (iNKT); small and medium-sized enterprises (SMEs); β-barrel assembly machinery (BAM); outer membrane protein (OMP).
